# Improved Inpatient Care through Greater Patient–Doctor Contact under the Hospitalist Management Approach: A Real-Time Assessment

**DOI:** 10.3390/ijerph18115718

**Published:** 2021-05-26

**Authors:** Wonjeong Chae, Dong-Woo Choi, Eun-Cheol Park, Sung-In Jang

**Affiliations:** 1 BK21 FOUR R&E Center for Precision Public Health, College of Health Science, Korea University, Seoul 02841, Korea; wjchae0816@korea.ac.kr; 2 Institute of Health Services Research, Yonsei University, Seoul 03722, Korea; CDW6027@yuhs.ac (D.-W.C.); ecpark@yuhs.ac (E.-C.P.); 3 Department of Preventive Medicine, College of Medicine, Yonsei University, Seoul 03722, Korea

**Keywords:** patient–doctor contact, patient–doctor contact frequency, contact duration, inpatient care, patient care quality

## Abstract

Objective: To examine the difference between hospitalist and non-hospitalist frequency of patient–doctor contact, duration of contact, cumulative contact time, and the amount of time taken by the doctor to resolve an issue in response to a medical call. Research Design and Measures: Data from 18 facilities and 36 wards (18 hospitalist wards and 18 non-hospitalist wards) were collected. The patient–doctor contact slip and medical call response slips were given to each inpatient ward to record. A total of 28,926 contacts occurred with 2990 patients, and a total of 8435 medical call responses occurred with 3329 patients. Multivariate logistic regression analyses and regression analyses were used for statistical analyses. Results: The average frequency of patient–doctor contact during a hospital stay was 10.0 times per patient for hospitalist patients. Using regression analyses, hospitalist patients had more contact with the attending physician (β = 5.6, standard error (SE) = 0.28, *p* < 0.0001). Based on cumulative contact time, hospitalists spent significantly more time with the patient (β = 32.29, SE = 1.54, *p* < 0.0001). After a medical call to resolve the issue, doctors who took longer than 10 min were 4.14 times (95% CI 3.15–5.44) and those who took longer than 30 min were 4.96 times (95% CI 2.75–8.95) more likely to be non-hospitalists than hospitalists. Conclusion: This study found that hospitalists devoted more time to having frequent encounters with patients. Therefore, inpatient care by a hospitalist who manages inpatient care from admission to discharge could improve the care quality.

## 1. Introduction

Two elements are essential to improve healthcare quality and patient outcomes. One is easy access to healthcare, and the other one is a positive patient–doctor relationship [1,2,3]. The frequency and duration of contact between the patient and doctor are measures of healthcare access as well as the patient–doctor relationship.

Studies of the patient–doctor relationship, such as patient–doctor contact, have focused on its association with patient outcomes including survival rate, pain management, and chronic disease management [4,5,6,7,8,9]. Although some studies associated these outcomes with patient–doctor contact, others did not [4,10]. In studies where positive relationships were reported, patients had an opportunity to be involved in their plan of care and receive education about their treatment [11,12,13]. These care conditions contribute to establishing a solid rapport between patients and doctors [7,8,12,13,14,15] and are linked to improved quality of care, patient safety, and satisfaction, including reduced medical errors and mortality [3,4,5,7,12,13]. These results are expected in both outpatient and inpatient care settings.

Traditionally, inpatient care is managed by a general internist or medical resident [16]. For inpatients, contact with doctors occurs during medical rounds; however, patients do not have round-the-clock access to their doctors, and patients who wish to meet with their attending physician usually find it difficult to reach them immediately [16,17,18]. A doctor has limited time to spend with patients due to their busy schedules and heavy workloads. Even when ad hoc consultation does occur, the contact time is often insufficient, especially from the patients’ perspective [18,19] and the hospital patient must wait to see a doctor.

The increasing aging population has increased the demand for geriatric care. Since elderly patients suffer from multiple diseases, they are admitted to the hospital for longer times and more often. Moreover, some diseases associated with aging require specialized care to prevent complications and adverse effects. The geriatric patient with multimorbidity is at risk of becoming a polypharmacy recipient [20,21]. If the patient receives various drugs from doctors, some prescriptions may be for unnecessary drugs [22]. Since the polypharmacy has become a public health issue, strategies to manage prescriptions among elderly patients should be implemented [20,22]. It is essential to have a doctor in charge of patient care even after admission. During the hospital stay, communication between the patient, patient’s family, and the doctor could affect the health outcome, making communication a critical element in health quality and patient safety [12,23]. Thus, having a doctor who can provide specialized care with an overview of healthcare would be beneficial to the patient [23,24].

To enhance the quality of care in the inpatient setting, the role of a hospitalist was first introduced in the United States and later expanded to other countries [16,25]. The hospitalist is a doctor in charge of managing inpatient care from admission to discharge. Since hospitalists work near hospitalized patients, these patients experience greater patient–doctor interaction [14,16,26]. The hospitalist system in Korea was implemented in 2016, featuring two unique criteria: hospitalists must be medical specialists and must stay in, or at least near, the hospitalist ward [27,28,29]. The increased level of patient–doctor contact provided by this new system is expected to improve the quality of care [28,29,30,31]. Therefore, we conducted a study to examine whether the frequency of patient–doctor contact, duration of contact, and cumulative contact time differed between hospitalists and non-hospitalists. In addition, we monitored the amount of time taken by a doctor to resolve an issue in response to a medical call. These measures of enhanced patient access to their doctors will help address whether the hospitalist system has improved inpatient care management.

## 2. Methods

### 2.1. Data Source

Data for this study were collected between September 2017 and December 2017 from the Health Insurance Review and Assessment Service (HIRA) Korea to evaluate the hospitalist policy in 18 facilities that participated in the Korean hospitalist pilot study. Participating facilities had a hospitalist ward as the case group. Hospitalists managed the hospitalist ward; however, patients also had contact with doctors in training (medical residents and interns) and other doctors as needed. The control group was managed by doctors in training and other doctors. The case and control groups were selected within the admitting medical department (internal medicine or surgery) from 18 facilities that all participated in hospitals for the beginning of Korean hospitalist system implementation. Thus, we collected patients from the same region, hospital level, and medical department to obtain study population homogeneity.

### 2.2. Measures

#### 2.2.1. Patient–Doctor Contact

Each time patient–doctor contact occurred, the doctor who made contact was required to complete a patient–doctor contact slip (a) which was placed at the patient’s bedside or at the nurses’ station. Information recorded on the slip included patient identification number for the study, shift (day or night) during which the contact occurred, purpose of the contact (procedure; condition check; physician rounds; requested referral; consultation, including explanation, prescription, consent; or any other reason), contacting duration, and role of the contact doctor (hospitalist, doctor in training, or other doctors). A total of 28,926 contacts were recorded with 4277 patients (Supplementary Table S1). Data from 2990 patients were used in this study, after being successfully linked to HIRA claim data for patient severity adjustments.

#### 2.2.2. Medical Call Response

A nurse was required to complete a medical call response slip (b) each time they summoned a doctor to see a patient. The information recorded on the slip included patient identification number for the study, contact time (day shift, night shift), contact doctor (hospitalist, doctor in training, or other doctors), purpose of the medical call (same as the patient–doctor contact slip), whether the call was answered, and response time after the call (or N/A, if the doctor did not answer). A total of 8435 slips were collected from 3329 patients.

(a) Example of patient–doctor contact recording slip

**Patient ID**

**Time “**

**Purpose**

**Duration**

**Contact Doctor**
1: Day 2: Night1: procedure; 2: condition check; 3: rounding; 4: requested referral; 5: consultation including explanation, prescription, consent; 6: othersMins1: hospitalist; 2: training doctor *; 3: other doctor1a23c014511a55441361,3“ Day (06:00–21:59), night (22:00–05:59); * training doctors: intern or resident doctor.

(b) Example of medical call response slip

**Patient ID**

**Time “**

**Contact Doctor**

**Purpose**

**Contact**

**Response Time**

**after the Call**
1: Day 2: Night1: hospitalist; 2: training doctor *; 3: other doctor1: procedure; 2: condition check; 3: rounding; 4: requested referral; 5: consultation including explanation, prescription, consent; 6: others1: succeed 2: Not succeedMins1a23c0115121a554412125“ Day (06:00–21:59), night (22:00–05:59); * training doctors: intern or resident doctor.

### 2.3. Covariates

Potentially confounding variables that were adjusted for in this study included sex, age, medical division (internal medicine, surgery), admission type (general admission, emergency room admission), shift type (day, night), region (capital, rural), and patient’s severity as determined by Charlson’s Comorbidity Index (CCI) score. Participating hospitals were also considered a potential confounding variable; however, we did not disclose such information in this report.

### 2.4. Statistical Analyses

General characteristics of the study population, distributions of contact frequency, and time were compared between the groups using *t*-tests and ANOVA. Medical call response data were analyzed using Chi-squared tests. To compare the frequency of patient–doctor contact and response time to a medical call between hospitalists (case) and non-hospitalists (control), we performed multivariable logistic regression analyses and multivariable regression analyses, adjusting all covariates. Results were considered statistically significant at *p* < 0.05. Statistical analyses were performed using SAS version 9.4 (SAS Institute, Cary, NC, USA).

### 2.5. Ethical Statement

Deidentified data were collected by the Health Insurance Review and Assessment Service to evaluate the policy. The data used in this study were exempted from Institutional Review Board review based on their classification as secondary data.

## 3. Results

### 3.1. Association of the Frequency of Patient–Doctor Contact

For all patients in the study, the average frequency of patient–doctor contact during a hospital stay was 7.3 times per patient (Table 1). In the case group, patients in hospitalist wards interacted with doctors an average of 10.0 times per patient during the hospital stay, compared to 4.2 interactions per patient in the control group (*p* < 0.001). This difference in contact frequency was observed across multiple medical divisions. Internal medicine patients, had 9.4 and 4.6 contacts per patient in the case and control groups, respectively (*p* < 0.001). In surgery, the case and control groups had 11.9 and 2.9 contacts per patient, respectively (*p* < 0.001). Significant differences in contact frequency were also observed across multiple service regions. In the capital area, the case and control groups had 8.7 and 4.2 contacts per patient, respectively (*p* < 0.001). Likewise, in rural areas, the case and control groups had 14.1 and 3.9 contacts per patient, respectively (*p* < 0.001). Supplementary Table S2 shows general characteristics of patients in the patient–doctor contact slips.

The results of regression analysis of the frequency of patient–doctor contact during hospital stays are presented in Table 1. The case group had significantly more contacts than did the control group (β = 5.6, standard error (SE) = 0.3, *p* < 0.001). When considering medical division and region, the case group had a significantly higher frequency of patient–doctor contacts than did the control group: internal medicine (β = 4.5, SE = 0.3, *p* < 0.001); surgery (β = 8.7, SE = 0.5, *p* < 0.001); capital area (β = 4.4, SE = 0.3, *p* < 0.001); and rural area (β = 10.1, SE = 0.8, *p* < 0.001).

### 3.2. Patient–Doctor Contact Duration Per Session

The overall average duration of patient–doctor contact was 4.9 min per session (Table 2). With respect to discipline, hospitalists averaged 4.7 ± 3.7 min per session (mean ± standard deviation [SD]), while training doctors and other doctors spent 5.0 ± 4.5 min and 5.6 ± 7.6 min per contact, respectively. Thus, hospitalists had the shortest contact duration, which was statistically significant (*p* < 0.001). This was also the case for specific contact purposes, such as procedure (mean ± SD = 8.8 ± 7.7 min for hospitalists, 9.4 ± 6.6 min for doctors in training, 27.7 ± 16.4 min for other doctors; *p* < 0.001) and condition check (4.4 ± 3.2 min for hospitalists, 4.4 ± 3.4 min for training doctors, 7.1± 8.1 min for other doctors; *p* < 0.001). 

Table 3 shows the result of adjusted regression analysis on the duration of patient contact per session. Compared to hospitalists, training doctors had spent shorter on average (β = −1.23, SE = 0.06, *p* < 0.001), and other doctors spend a similar amount of time on average (β = 0.44, SE = 0.09, *p* < 0.001).

### 3.3. Association of Cumulative Contact Time with the Purpose of Patient–Doctor Contact

The average total cumulative contact time was 51.3 min for the case group and 17.6 min for the control group (Supplementary Table S3), which is a statistically significant difference (β = 32.29, SE = 1.54, *p* < 0.001; Table 4). When examined by purpose of contact, the cumulative contact time with doctors was also significantly longer for the case group than the control group (physician rounds: β = 16.38, SE = 0.71, *p* < 0.001; consultation: β = 6.53, SE = 0.35, *p* < 0.001; condition check: β = 5.82, SE = 0.46, *p* < 0.001; Supplementary Table S3). Further subgroup analyses by medical division or region showed similar trends of longer cumulative doctor contact times with patients in the case group: internal medicine department (total β = 25.76, SE = 1.54, *p* < 0.001); surgery (total β = 52.42, SE = 3.34, *p* < 0.001); capital area (total β = 26.62, SE = 1.64, *p* < 0.001); rural area (consultation β = 9.07, SE = 0.80, *p* < 0.001).

### 3.4. Response to a Medical Call and Resolving the Issue

The general characteristics of information collected using medical call slips are presented in Supplementary Table S4. In response to calls and resolving the issue, non-hospitalists took more time than hospitalists did (Table 5, Supplementary Table S5). The time after a medical call to doctors by the nurse and resolve the issue was measured in four categories: exceeding 10 min, exceeding 30 min, exceeding 60 min, and exceeding 120 min reference to less than 10 min. As the results, non-hospitalists took longer in response to a medical call by 4.14 times (odds ratio (OR) 4.14, 95% confidence interval (CI) 3.15–5.44), 4.96 times (OR 4.96, CI 2.75–8.95), 5.06 times (OR 5.06, CI 1.73–14.78), and 6.07 (OR 6.07, CI 0.66–55.76) times more likely to take more than were hospitalists, accordingly. 

**Figure 1:**
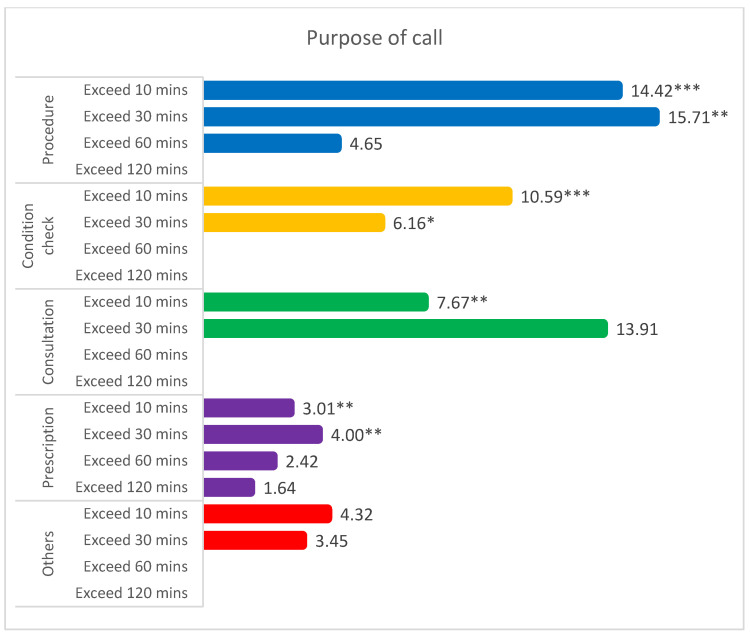
Results of subgroup analysis on the time to response a medical call of non-hospitalist compared to hospitalists: purpose of a medical call (reference: hospitalist, less than 10 min; shown in odds ratio). *** *p* < 0.001, ** *p* < 0.01, * *p* < 0.05.

Sub-group analyses on purpose of the medical call were conducted (Figure 1). As for the purpose of a medical call, non-hospitalists were 14.42 times (OR 14.42, CI 4.51–431.13) more likely to take over 10 min to respond to a medical call and resolve the issue and 15.71 times (OR: 15.71, CI: 2.42–102.15) more likely to take over 30 min to respond to a medical call and resolve the issue. Sub-group analyses on medical division, admission type, region, and shift were conducted, and results showed that non-hospitalist took longer to response to a medical call and resolved the issue (Supplementary Figure S1). 

## 4. Discussion

Hospital patients require constant care and close monitoring because their conditions can be unpredictable. To receive sufficient care during hospital stays, inpatients should have access to doctors because access to doctors is known to improve patient safety [5]. Therefore, more frequent contact and longer contact time with doctors can improve the quality of patient care and safety in inpatient care settings [8,32,33].

The Korean hospitalist system was established with the purpose of enhancing inpatient safety and quality of care [27,28,29]. As part of the policy evaluation of the system, data were available from 18 facilities, including 36 wards, that participated in the initial hospitalist pilot program. We used this data to compare the frequency of patient–doctor contact, cumulative contact time, and time per contact within 18 hospitalist and 18 non-hospitalist wards [29]. The results of our study support that the hospitalist system has a positive impact to improve inpatient healthcare quality in Korea.

Our study showed that patients in hospitalist wards had more contact with doctors than patients in control wards, indicating that patients in hospitalist wards had more access to doctors during their hospital stay. Additionally, hospitalist wards reported a longer cumulative time of patient–doctor contact than in non-hospitalist wards, especially when the purpose of contact was for a condition check, a consultation, or even during rounds. In contrast, the length of time per contact was shorter for hospitalists compared to other doctors. Our interpretation is that because contact occurs more frequently in hospitalist wards, prolonged contact is less necessary at each patient–doctor encounter. In the event of a medical call, hospitalists responded more quickly and took less time to resolve an issue than other doctors. During hospitalization, nurses summon a doctor when the patient requires a doctor’s attention. When this happens, how quickly the doctor responds to the nurse’s call to provide care could certainly affect patient outcomes, especially in an emergency. In addition, this study found that a hospitalist’s response to medical calls resulted in them being able to resolve the issue more quickly, perhaps because they stayed in the ward near the patient. Our analysis suggests that hospitalists are more accessible to patients and can maintain patient safety and deliver high-quality care to patients during hospital stays.

The study is conducted based on the evaluation of the Korean hospitalist system. The Korean hospitalist system was implemented in 2016 with support from the government, medical professionals, and patients [29]. Prior to the hospitalist system, the majority of inpatient care was given by medical residents; under the hospital’s heavy workload, care quality and patient safety concerns remained. Furthermore, due to the medical insurance scheme for inpatient care, patients could not see the doctor as much as they wanted. As the hospital can claim and reimburse the inpatient care under only an inpatient care fee, which includes the entire care that occurred in a day, the number of times patients can see a doctor is limited and short [27,29]. Patients wanted to have care from the specialist other than residents have more encounters with a specialist and receive inpatient care service by a specialist. Therefore, Korea implemented the Korean Hospitalist system to be suitable for the existing medical system. As Korea is under the universal health insurance system, the system had to implement the institutional level by the government [27,29].

The data for this study were collected under the government’s supervision to evaluate the Korean hospitalist system. In the hospitalist ward, patients had more contact with doctors, hospitalists, and doctors quickly in response to a medical call. The findings support the concept of increased healthcare quality in inpatient care during hospital stays, as hospitalist wards showed a higher number of patient–doctor encounters and quicker response to a medical call. The more frequent contact with the patient leads to understanding the patient’s condition, and quicker response allows to lower the potential risk of the patient. Moreover, better access to medical specialists may influence not only patient outcomes but also increase patient satisfaction during hospital stays [12,17,24,31,32,33]. More contact with doctors allows patients to develop trust, which may enhance the patient–doctor relationship. In addition, when a doctor devotes more time to a patient, quality of care and patient safety are also expected to improve [5,7,15,17,30,31,32,33,34]; as an example, the recent studies conducted regarding Korean hospitalists showed that hospitalists have a positive health outcome as they take care of patients 24/7 [29,34].

### Limitations and Strengths

Our study has several strengths. First, it is the first study to compile real-world data on the frequency of patient–doctor contact, cumulative contact time, and duration per contact for patients in Korean facilities. Second, a case-control study design was used with a large sample size. Data were collected from all hospitals that had implemented the hospitalist system. Third, not only did we measure the number of patient–doctor contacts, but we also evaluated cumulative contact time and duration per contact. Moreover, we measured the time taken by doctors to solve problems when responding to medical calls.

Despite these strengths, possible limitations should be addressed. First, the limitation could be found in data and study design. The data were obtained directly from doctors’ or nurses’ handwritten observations, which could potentially include inaccuracies. More precise means of collecting the data would be helpful for future studies. As per the study design, we conducted the study as a case-control study; however, the sampling of the case group and control group at the baseline does not meet case-control design requirements. The study is a quasi-experimental study to evaluate the recent implementation of the hospitalist system in Korea. Thus, after the new system’s firm establishment, the study should be conducted again with more in-depth data and study design. Second, most hospitals providing the data were tertiary hospitals that could represent the general patient population. In addition, study data were categorized on the basis of the ward, i.e., from a hospitalist ward (case) or non-hospitalist ward (control). As a result, patient characteristics were not completely uniform between the groups. In addition, we did not consider the potential for confounding factors due to transferring a patient from one ward to another that reduce study bias, and we adjusted for medical division and patient severity. Moreover, although the reason for admission may have differed among hospitals, inpatient hospital care should be similar. Third, the average length of stay in the case and control groups was different (case: 6.5 days, SD 6.1 vs. control: 5.7 days, SD 5.3, *p* = 0.0116). For this study, length of stay was not included in the analysis, which is a limitation of the study. During the hospital stay, patients were allowed to move from one ward to another; some patients were transferred from the non-hospitalist ward to the hospitalist ward after the initial admission; even we were able to obtain the patient’s total length of stay. Therefore, to overcome the limitation, the analysis was conducted with an adjusted model including age and Charlson Comorbidity Index score. The study was conducted based on the pilot study of the new healthcare system. After expanding the Korean hospitalist system, the length of stay should be studied further.

## 5. Conclusions

This study investigated differences in the frequency of patient–doctor contact, cumulative contact time, and time per contact between patients in hospitalist and non-hospitalist wards to evaluate the Korean hospitalist system, which was recently implemented. As the hospitalist wards are managed by a specialist 24/7, hospitalist ward patients had more frequent contact with doctors and spent more time. The finding describes the process of improving health quality in Donabedian’s health quality evaluation that the hospitalist care could contribute to solving inpatient care problem in Korea. Thus, to improve hospital inpatient care management, implementing the hospitalist system showed positive feedback that Korea is expanding the hospitalist system from its initial 18 facilities. The implementation was acquired from the great need for better inpatient service under the Korean healthcare system by patients, medical professionals, and the government. Those countries seeking to enhance the healthcare system could consider adopting hospitalist with modification to fit the existing healthcare system of the country.

## Figures and Tables

**Table 1:** General characteristics of the frequency of patient–doctor contact during the hospital stays and results of regression analysis ⁺.

Variables	Total			Case (Hospitalist Ward)			Control (Non-Hospitalist Ward)			*p*-Value	Adjusted Difference ⁺	
	Mean	±	SD	Mean	±	SD	Mean	±	SD		β	SE
Total	7.3	±	9.0	10.1	±	11.0	4.2	±	4.0	<0.001	5.6 ***	0.3
Sex												
Male	7.0	±	8.2	9.7	±	10.0	4.2	±	4.2	<0.001	5.5 ***	0.4
Female	7.7	±	9.8	10.4	±	11.8	4.1	±	3.7	<0.001	5.7 ***	0.4
Age												
19 and below	6.0	±	5.8	7.9	±	7.7	4.3	±	2.5	0.080	1.6 *	0.8
20–29	6.1	±	6.6	9.3	±	8.7	4.2	±	3.8	<0.001	3.9 *	1.6
30–39	6.3	±	6.3	8.4	±	7.2	3.7	±	3.6	<0.001	4.6 ***	1.0
40–49	6.5	±	5.7	8.4	±	6.3	4.0	±	3.4	<0.001	4.0 ***	0.5
50–59	7.1	±	9.1	9.8	±	11.3	3.8	±	3.2	<0.001	5.6 ***	0.6
60–69	7.7	±	9.9	10.8	±	12.3	4.1	±	3.6	<0.001	6.7 ***	0.6
70–79	7.8	±	9.9	10.9	±	12.3	4.3	±	4.0	<0.001	6.3 ***	0.6
80 and above	8.6	±	10.6	11.0	±	12.1	5.4	±	7.0	<0.001	4.5 ***	1.1
Medical division												
Internal medicine	7.2	±	9.2	9.4	±	11.4	4.6	±	4.2	<0.001	4.5 ***	0.3
Surgery	7.8	±	8.4	11.9	±	9.3	2.9	±	3.0	<0.001	8.7 ***	0.5
Admission type												
General admission	7.7	±	9.4	10.7	±	11.4	4.2	±	4.1	<0.001	6.2 ***	0.3
ER admission	4.1	±	2.7	4.5	±	3.1	3.8	±	2.3	0.017	1.0 **	0.3
Region												
Capital	6.6	±	7.1	8.7	±	8.3	4.2	±	4.3	<0.001	4.4 ***	0.3
Rural	9.8	±	13.1	14.1	±	15.8	3.9	±	2.5	<0.001	10.1 ***	0.8
CCI ^‡^												
0	6.0	±	7.5	8.2	±	9.9	4.1	±	3.7	<0.001	3.4 ***	0.5
1	8.1	±	10.2	10.0	±	13.0	5.9	±	4.9	<0.001	3.2 ***	0.9
2	6.9	±	7.6	9.3	±	8.8	3.9	±	4.1	<0.001	5.7 ***	0.4
3	10.0	±	11.8	13.7	±	13.4	3.6	±	2.9	<0.001	9.4 ***	1.2
4<	9.9	±	12.9	14.1	±	15.1	3.7	±	3.2	<0.001	10.2 ***	1.1

⁺ Fully adjusted model (adjusted: sex, age, medical division, admission type, region, shift type, CCI) of difference in contact frequency reference to control (non-hospitalist ward). *** *p* < 0.001, ** *p* < 0.01, * *p* < 0.05; ^‡^ Charlson’s Comorbidity Index.

**Table 2:** Average of patient–doctor contact duration per session.

Variables	Contact Time (min)												
	Total			Hospitalist			Training Doctor			Other Doctor			*p*-Value
	Mean	±	SD	Mean	±	SD	Mean	±	SD	Mean	±	SD	
Total													
	4.9	±	4.5	4.7	±	3.7	5.0	±	4.5	5.6	±	7.6	<0.001
Medical division													
Internal medicine	4.7	±	4.6	4.4	±	3.2	4.9	±	4.3	5.8	±	8.2	<0.001
Surgery	5.5	±	4.3	5.6	±	4.5	4.9	±	3.4	3.8	±	2.6	<0.001
Admission type													
General admission	4.5	±	3.8	4.7	±	3.7	4.4	±	4.1	3.8	±	4.2	<0.001
ER admission	9.2	±	8.2	7.4	±	4.1	8.0	±	3.6	13. 6	±	13.5	<0.001
Region													
Capital	5.1	±	4.8	5.0	±	3.9	4.4	±	3.9	8.3	±	9.4	<0.001
Rural	4.5	±	4.03	4.1	±	3.1	7.5	±	5.7	3.3	±	4.5	<0.001
Shift													
Day	4.9	±	4.5	4.7	±	3.6	4.9	±	4.5	5.6	±	7.6	<0.001
Night	5.8	±	4.3	7.5	±	5.0	6.2	±	5.3	5.8	±	2.9	0.196
Purpose of the contacts													
Procedure	11.0	±	10.3	8.8	±	7.7	9.4	±	6.6	27.7	±	16.4	<0.001
Condition check	4.5	±	3.3	4.4	±	3.2	4.4	±	3.4	7.0	±	8.1	<0.001
Rounding	3.8	±	2.2	4.2	±	2.2	3.1	±	2.5	3.0	±	2.1	<0.001
Consultation with other doctors	9.3	±	5.8	9.2	±	10.1	9.3	±	6.1	9.4	±	6.2	0.996
Consultation with patients	7.7	±	5.8	7.9	±	6.4	7.7	±	5.0	9.4	±	8.8	0.099
Others	6.1	±	7.9	5.5	±	7.7	5.1	±	5.7	17.5	±	17.7	0.079

**Table 3:** Regression results of patient–doctor contact duration per session *.

Variables	Patient–Doctor Contact Duration Per Session (min)					
	Training Doctor			Other Doctor		
	β	SE	*p*-Value	β	SE	*p*-Value
Total						
	−1.23	0.06	<0.001	0.44	0.09	<0.001
Medical division						
Internal medicine	−1.70	0.07	<0.001	0.25	0.09	0.004
Surgery	−0.50	0.13	<0.001	−1.10	0.46	0.017
Admission type						
General admission	−0.70	0.06	<0.001	−0.97	0.08	<0.001
ER admission	−3.56	0.43	<0.001	5.93	0.52	<0.001
Region						
Capital	−1.72	0.07	<0.001	3.40	0.15	<0.001
Rural	1.38	0.12	<0.001	−1.71	0.09	<0.001
Shift						
Day	−1.21	0.12	<0.001	0.43	0.09	<0.001
Night	−2.87	0.59	<0.001	−3.81	1.50	0.011
Purpose of the contacts						
Procedure	−2.35	0.60	<0.001	15.22	1.01	<0.001
Condition check	−1.65	0.11	<0.001	1.25	0.28	<0.001
Rounding	−1.47	0.04	<0.001	−0.95	0.05	<0.001
Consultation with other doctors	1.28	1.23	0.301	−0.01	0.99	0.994
Consultation with patients	−0.57	0.24	0.017	0.72	0.84	0.386
Others	−3.15	6.11	0.611	23.78	7.55	0.004

* Fully adjusted model (adjusted: sex, age, medical division, admission type, region, shift type, CCI). Relative to non-hospitalist ward (reference: non-hospitalist ward).

**Table 4:** Results of regression analysis on cumulative contact time by the purpose of patient–doctor contact during the hospital stays ⁺.

Variables	Cumulative Contact Time (min)		
	Hospitalist Ward		*p*-Value
	β	SE	
Cumulative contact time			
Total	32.29	1.54	<0.001
Procedure	2.39	0.50	<0.001
Condition check	5.82	0.46	<0.001
Rounding	16.38	0.71	<0.001
Consultation with other doctors	1.27	0.24	<0.001
Consultation with patients	6.53	0.35	<0.001
Others	0.10	0.05	0.035
Medical division			
Internal medicine			
Total	25.76	1.67	<0.001
Procedure	1.25	0.55	0.024
Condition check	4.39	0.46	<0.001
Rounding	13.50	0.78	<0.001
Consultation with other doctors	1.70	0.31	<0.001
Consultation with patients	4.95	0.39	<0.001
Others	0.13	0.06	0.025
Surgery			
Total	52.42	3.63	<0.001
Procedure	6.05	1.15	<0.001
Condition check	10.32	1.39	<0.001
Rounding	25.42	1.63	<0.001
Consultation with other doctors	−0.04	0.03	0.165
Consultation with patients	10.99	0.76	<0.001
Others	−0.02	0.06	0.717
Region			
Capital area			
Total	26.62	1.64	<0.001
Procedure	1.08	0.52	0.038
Condition check	4.00	0.49	<0.001
Rounding	16.06	0.88	<0.001
Consultation with other doctors	0.14	0.20	0.4898
Consultation with patients	5.61	0.39	<0.001
Others	0.13	0.06	0.037
Rural area			
Total	53.35	3.78	<0.001
Procedure	6.44	1.28	<0.001
Condition check	11.89	1.16	<0.001
Rounding	20.44	1.13	<0.001
Consultation with other doctors	5.41	0.81	<0.001
Consultation with patients	9.07	0.80	<0.001
Others	0.00	0.01	0.608

⁺ Fully adjusted model (adjusted: sex, age, medical division, admission type, region, shift type, CCI). Relative to non-hospitalist ward (reference: non-hospitalist ward).

**Table 5:** Results of logistic regression analysis on the time to response a medical call of non-hospitalist compared to hospitalists ⁺.

Variable	Non-Hospitalist		
	OR	95%CI	*p*-Value
Exceed 10 mins	4.14	(3.15–5.44)	<0.001
Exceed 30 mins	4.96	(2.75–8.95)	<0.001
Exceed 60 mins	5.06	(1.73–14.78)	0.003
Exceed 120 mins	6.07	(0.66–55.76)	0.111

**⁺** Reference: hospitalist, less than 10 mins.

## Data Availability

Data are available from the authors upon reasonable request and with permission of the Health Insurance Review and Assessment Service (HIRA).

## Supplementary Materials

The following are available online at https://www.mdpi.com/article/10.3390/ijerph18115718/s1, Figure S1: Results of subgroup analysis on the time to response a medical call of non-hospitalist compared to hospitalists: medical division, admission type, region, and shift type, Table S1: The distribution of patient-doctor contacts, Table S2: General characteristics of patients who were in the contact recording slips, Table S3: Average of cumulative contact time for case and control group, Table S4: General characteristics and distribution of the medical call response slips, Table S5: Results of logistic regression analysis on the time to response a medical call compares to hospitalists.
