# Supplementary material for: Improved Inpatient Care through Greater Patient–Doctor Contact under the Hospitalist Management Approach: A Real-Time Assessment
Source: Int J Environ Res Public Health. 2021 May 26;18(11):5718. doi: 10.3390/ijerph18115718 (PMC8198090; doi:10.3390/ijerph18115718)
Supplement: Supplementary file 1 [file ijerph-18-05718-s001.zip › ijerph-1194376-supplementary.pdf]

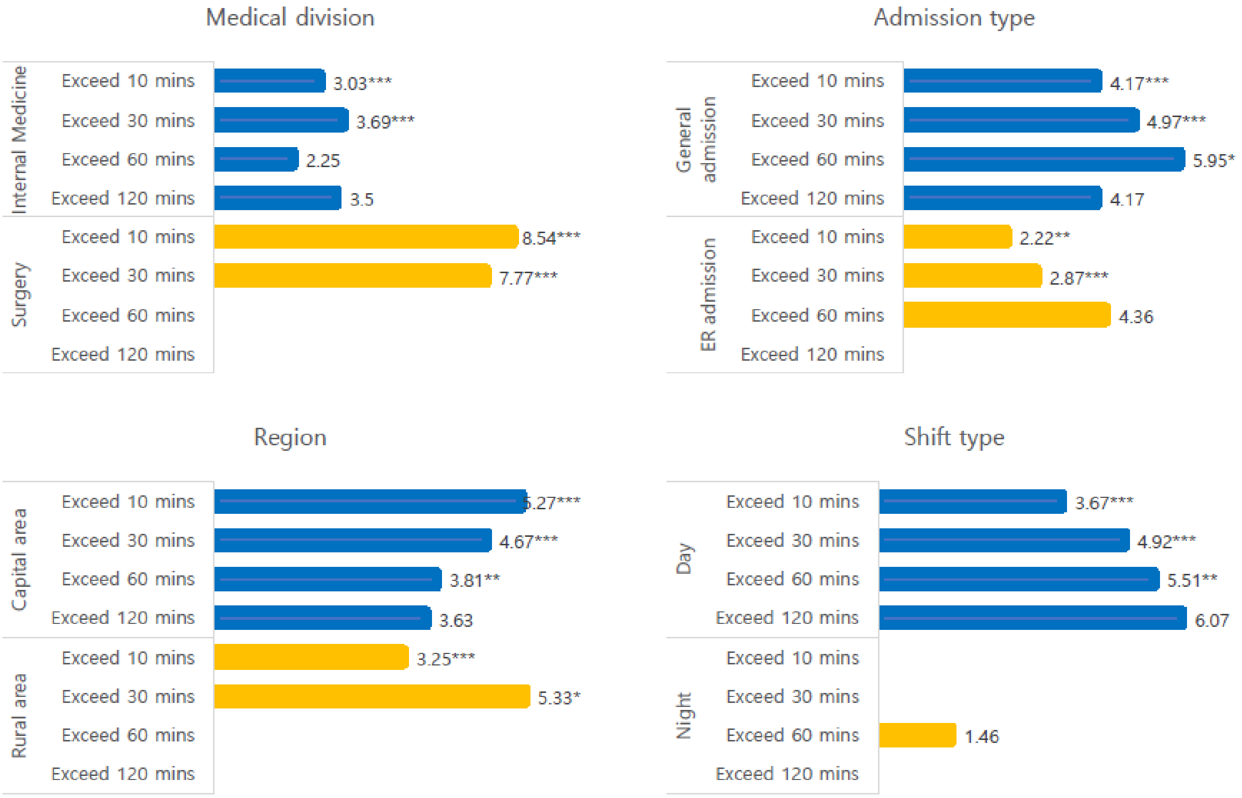

Figure S1. Results of subgroup analysis on the time to response a medical call of non-hospitalist compared to hospitalists: medical division, admission type, region, and shift type (reference: hospitalist, less than 10 mins; shown in odds ratio). *** $p < 0.001$, ** $p < 0.01$, * $p < 0.05$.

Table S1. The distribution of patient-doctor contacts.

**Variables**	**Total**		**Case**						**Control**		***p*-Value**
			**Hospitalist**		**Training Doctors**		**Other Doctors**				
	N	%	*N*	%	*N*	%	*N*	%	*N*	%	
Number of patients	4277	100.0			2167	50.7%			2110	49.3%	
Total contact	28926	100.0	17746	61.3	1269	4.4	1014	3.5	8897	30.8	
Admitted Department											
Internal medicine	17325	73.2	10947	63.2	1179	6.8	983	5.7	4216	24.3	<.0001
Surgery	6329	26.8	5187	82.0	28	0.4	3	0.0	1111	17.6	
Admission Type											
General admission	21893	92.6	15556	71.1	896	4.1	754	3.4	4687	21.4	<.0001
ER admission	1761	7.4	578	32.8	311	17.7	232	13.2	640	36.3	
Area											
Capital	18154	69.1	11719	64.6	822	4.5	338	1.9	5275	29.1	<.0001
Rural	8136	30.9	6027	74.1	447	5.5	676	8.3	986	12.1	
Shift											
Day	25851	98.3	17685	68.4	1097	4.2	1010	3.9	6059	23.4	<.0001
Night	435	1.7	57	13.1	172	39.5	4	0.9	202	46.4	
Contact doctor											
Hospitalist	17829	61.6	17746	99.5		0.0		0.0	83	0.5	<.0001
Training doctors	7447	25.7		0.0	1269	17.0		0.0	6178	83.0	
Other doctors	3647	12.6		0.0		0.0	1014	27.8	2633	72.2	
Purpose of contact †											
Procedure	1696	6.2	618	36.4	486	28.7	57	3.4	535	31.5	<.0001
Condition check	6040	22.1	4145	68.6	164	2.7	23	0.4	1708	28.3	
Rounding	15627	57.3	11308	72.4	144	0.9	539	3.4	3636	23.3	
Consultation with other doctors	440	1.6	20	4.5	15	3.4	378	85.9	27	6.1	
Consultation with patients	3424	12.6	1972	57.6	499	14.6	14	0.4	939	27.4	
Others	46	0.2	25	54.3	6	13.0	1	2.2	14	30.4	
Contact time (mins)											

Less than 1	101	0.4	1	1.0	14	13.9	0	0.0	86	85.1	<.0001
More than 1, less than 5	13163	46.0	7836	59.5	267	2.0	404	3.1	4656	35.4	
More than 5, less than 10	10890	38.1	7236	66.4	438	4.0	232	2.1	2984	27.4	
More than 10, less than 20	3568	12.5	2205	61.8	361	10.1	206	5.8	796	22.3	
More than 20	878	3.1	324	36.9	185	21.1	109	12.4	260	29.6	

† Able to mark more than one.

Table S2. General characteristics of patients who were in the contact recording slips.

Variables	Total		Case		Control		*p*-Value
	N	%	*N*	%	*N*	%	
Total	2990	100	1610	53.8	1380	46.2	<.0001
Sex							
Male	2242	75.0	782	34.9	758	33.8	0.0005
Female	748	25.0	828	110.7	622	83.2	
Age							
19 and below	32	1.1	15	46.9	17	53.1	0.0294
20–29	103	3.4	39	37.9	64	62.1	
30–39	199	6.7	107	53.8	92	46.2	
40–49	385	12.9	222	57.7	163	42.3	
50–59	665	22.2	362	54.4	303	45.6	
60–69	702	23.5	373	53.1	329	46.9	
70–79	643	21.5	341	53.0	302	47.0	
80 and above	261	8.7	151	57.9	110	42.1	
Medical division							
Internal medicine	2242	75.0	1207	53.8	1035	46.2	
Surgery	748	25.0	403	53.9	345	46.1	
Admission type							
General admission	2652	88.7	1438	54.2	1214	45.8	0.4717
ER admission	338	11.3	172	50.9	166	49.1	
Region							
Capital	2279	76.2	1199	52.6	1080	47.4	0.0172
Rural	711	23.8	411	57.8	300	42.2	
CCI							
0	798	26.7	368	46.1	430	53.9	<.0001
1	323	10.8	170	52.6	153	47.4	
2	1347	45.1	754	56.0	593	44.0	
3	201	6.7	127	63.2	74	36.8	
4<	321	10.7	191	59.5	130	40.5	

Table S3. Average of cumulative contact time for case and control group †.

Variables	TOTAL			Case			Control			*p*-Value
	Mean	±	SD	Mean	±	SD	Mean	±	SD	
Cumulative contact time										
Total	35.8	±	49.6	51.3	±	60.6	17.6	±	20.9	<.0001
Procedure	4.9	±	14.9	6.1	±	17.8	3.5	±	10.4	0.0043
Condition check	7.0	±	14.4	9.8	±	17.9	3.6	±	7.4	<.0001
Rounding	17.0	±	22.7	24.7	±	26.9	8.0	±	11.0	<.0001
Consultation with other doctors	1.7	±	7.4	2.3	±	9.3	1.0	±	4.1	<.0001
Consultation with patients	6.8	±	11.1	10.0	±	13.2	3.0	±	6.2	<.0001
Others	0.1	±	1.3	0.1	±	1.8	0.0	±	0.4	0.0442
Medical division										
Internal medicine										
Total	33.7	±	47.4	46.3	±	58.4	18.9	±	21.9	<.0001
Procedure	4.7	±	14.9	5.4	±	17.3	3.9	±	11.3	0.0184
Condition check	6.0	±	12.8	8.2	±	15.9	3.4	±	6.7	<.0001
Rounding	15.4	±	21.2	21.8	±	25.3	7.9	±	10.8	<.0001
Consultation with other doctors	2.2	±	8.5	3.1	±	10.6	1.3	±	4.7	<.0001
Consultation with patients	6.5	±	10.8	9.0	±	12.8	3.6	±	6.6	<.0001
Others	0.1	±	1.5	0.1	±	2.0	0.0	±	0.1	0.0473
Surgery										
Total	42.1	±	55.2	66.3	±	64.3	13.7	±	16.8	<.0001
Procedure	5.4	±	15.1	8.1	±	19.2	2.2	±	6.8	<.0001
Condition check	10.0	±	18.2	14.8	±	22.2	4.4	±	9.3	<.0001
Rounding	22.0	±	26.1	33.5	±	29.3	8.5	±	11.7	<.0001

Consultation with other doctors	0.0	±	0.4	0.0	±	0.0	0.0	±	0.6	0.147
Consultation with patients	7.6	±	12.2	13.0	±	14.0	1.3	±	4.5	<.0001
Others	0.1	±	0.7	0.0	±	0.6	0.1	±	0.8	0.8677
Region										
Capital										
Total	32.7	±	43.5	45.7	±	52.9	18.4	±	22.2	<.0001
Procedure	4.4	±	12.9	5.0	±	14.3	3.8	±	11.2	0.0196
Condition check	6.1	±	12.9	8.1	±	16.0	3.9	±	7.7	<.0001
Rounding	17.2	±	23.5	24.9	±	28.2	8.8	±	11.9	<.0001
Consultation with other doctors	1.3	±	4.9	1.3	±	5.2	1.2	±	4.6	0.6411
Consultation with patients	5.7	±	10.4	8.5	±	12.7	2.6	±	5.8	<.0001
Others	0.1	±	1.5	0.1	±	2.1	0.0	±	0.4	0.0536
Rural										
Total	45.5	±	64.5	67.9	±	76.5	14.9	±	14.7	<.0001
Procedure	6.3	±	19.9	9.1	±	25.3	2.5	±	6.6	<.0001
Condition check	9.7	±	18.2	14.9	±	21.9	2.7	±	6.4	<.0001
Rounding	16.3	±	19.9	24.3	±	22.5	5.2	±	6.0	<.0001
Consultation with other doctors	3.0	±	12.3	5.2	±	15.8	0.2	±	1.4	<.0001
Consultation with patients	10.2	±	12.5	14.3	±	13.9	4.5	±	7.3	<.0001
Others	0.0	±	0.1	0.0	±	0.1	0.0	±	0.1	0.8099

† Fully adjusted analyses including hospitals (wards).

Table S4. General characteristics and distribution of the medical call response slips.

Variables	Total		Case				Control		*p*-Value
			Hospitalist		Non-Hospitalist		Non-Hospitalist		
	N	%	*N*	%	*N*	%	*N*	%	
Patients	3329		1691				1638		
Sex									0.0019
Male	4149	49.19	1446	34.85	915	22.05	1788	43.09	
Female	4286	50.81	1765	41.18	817	19.06	1704	39.76	
Age									<0.0001
19 and below	139	1.64	49	35.25	12	8.63	78	56.12	
20–29	265	3.13	67	25.28	55	20.75	143	53.96	
30–39	535	6.32	211	39.44	91	17.01	233	43.55	
40–49	914	10.80	452	49.45	183	20.02	279	30.53	
50–59	1809	21.38	718	39.69	341	18.85	750	41.46	
60–69	1858	21.96	730	39.29	403	21.69	725	39.02	
70–79	2010	23.75	723	35.97	425	21.14	862	42.89	
80 and above	932	11.01	271	29.08	232	24.89	429	46.03	
Medical division									<0.0001
Internal medicine	9006	77.32	2966	32.93	2026	22.50	4014	44.57	
Surgery	2641	22.68	1215	46.01	395	14.96	1031	39.04	
Admission type									<0.0001
General admission	8414	73.27	3418	40.62	1578	18.75	3418	40.62	
ER admission	3069	26.73	747	24.34	846	27.57	1476	48.09	
Region									<0.0001
Capital	9221	78.64	2944	31.93	2123	23.02	4154	45.05	
Rural	2504	21.36	1262	50.40	302	12.06	940	37.54	
Shift									<0.0001
Day	9780	83.43	4065	41.56	1539	15.74	4176	42.70	
Night	1942	16.57	139	7.16	886	45.62	917	47.22	
Called doctor									<0.0001
Hospitalists	4271	36.43	4206	98.48	0	0.00	65	1.52	
Training doctor	5144	43.87	0	0.00	219	4.26	4925	95.74	
Other doctors	2310	19.70	0	0.00	2206	95.50	104	4.50	
Purpose of the call †									<0.0001
Procedure	2050	16.57	669	32.63	429	20.93	952	46.44	
Condition check	5507	44.50	1867	33.90	1301	23.62	2339	42.47	
Consultation with patients	1039	8.40	663	63.81	110	10.59	266	25.60	
Prescription	2452	19.82	1114	45.43	417	17.01	921	37.56	
Others	1326	10.72	461	34.77	201	15.16	664	50.08	
Succeed to reach a doctor									<0.0001
Yes	11672	99.55	4195	35.94	2406	20.61	5071	43.45	
No	53	0.45	11	20.75	19	35.85	23	43.40	
Time to solve the problem after the call									0.0099

Less than 10 mins	11187	95.41	4116	36.79	2345	20.96	4726	42.25
Exceed 10 mins	375	3.20	72	19.20	54	14.40	249	66.40
Exceed 30 mins	104	0.89	11	10.58	17	16.35	76	73.08
Exceed 60 mins	31	0.26	5	16.13	4	12.90	22	70.97
Exceed 120 mins	28	0.24	2	7.14	5	17.86	21	75.00

† Multiple selection allowed.

Table 5. Results of logistic regression analysis on the time to response a medical call compares to hospitalists.

Variables		Non-Hospitalist		
	OR	95%CI		*p*-Value
Total				
Exceed 10 mins	4.14	(3.15 -	5.44)	<.001
Exceed 30 mins	4.96	(2.75 -	8.95)	<.001
Exceed 60 mins	5.06	(1.73 -	14.78)	0.003
Exceed 120 mins	6.07	(0.66 -	55.76)	0.111
Medical division				
Internal Medicine				
Exceed 10 mins	3.03	(2.15 -	4.29)	<.001
Exceed 30 mins	3.69	(1.74 -	7.83)	0.001
Exceed 60 mins	2.25	(0.52 -	9.80)	0.280
Exceed 120 mins	3.50	(0.39 -	31.63)	0.265
Surgery				
Exceed 10 mins	8.54	(4.53 -	16.11)	<.001
Exceed 30 mins	7.77	(2.80 -	21.59)	<.001
Exceed 60 mins				
Exceed 120 mins				
Admission type				
General admission				
Exceed 10 mins	4.17	(3.13 -	5.57)	<.001
Exceed 30 mins	4.97	(2.48 -	9.94)	<.001
Exceed 60 mins	5.95	(1.16 -	30.46)	0.032
Exceed 120 mins	4.17	(0.44 -	39.32)	0.212
ER admission				
Exceed 10 mins	2.22	(1.30 -	3.79)	0.003
Exceed 30 mins	2.87	(1.69 -	4.85)	<.001
Exceed 60 mins	4.36	(0.43 -	44.18)	0.213
Exceed 120 mins				
Region				
Capital area				
Exceed 10 mins	5.27	(3.50 -	7.93)	<.001
Exceed 30 mins	4.67	(2.38 -	9.16)	<.001
Exceed 60 mins	3.81	(1.23 -	11.76)	0.020
Exceed 120 mins	3.63	(0.32 -	41.37)	0.300
Rural area				
Exceed 10 mins	3.25	(1.99 -	5.29)	<.001
Exceed 30 mins	5.33	(1.20 -	23.67)	0.028
Exceed 60 mins				
Exceed 120 mins				
Shift type				
Day				
Exceed 10 mins	3.67	(2.74 -	4.90)	<.001
Exceed 30 mins	4.92	(2.57 -	9.43)	<.001
Exceed 60 mins	5.51	(1.51 -	20.12)	0.001
Exceed 120 mins	6.07	(0.66 -	55.77)	0.111
Night				
Exceed 10 mins				
Exceed 30 mins				
Exceed 60 mins	1.46	(0.73 -	2.92)	0.287
Exceed 120 mins				
Purpose of call				
Procedure				
Exceed 10 mins	14.42	(4.51 -	46.13)	<.001
Exceed 30 mins	15.71	(2.42 -	102.15)	0.004
Exceed 60 mins	4.65	(0.69 -	31.61)	0.116
Exceed 120 mins				

Condition check				
Exceed 10 mins	10.59	(4.73 -	23.70)	<.001
Exceed 30 mins	6.16	(1.47 -	25.82)	0.013
Exceed 60 mins				
Exceed 120 mins				
Consultation				
Exceed 10 mins	7.67	(2.29 -	25.71)	0.001
Exceed 30 mins	13.91	(0.77 -	252.65)	0.075
Exceed 60 mins				
Exceed 120 mins				
Prescription				
Exceed 10 mins	3.01	(1.72 -	5.26)	0.001
Exceed 30 mins	4.00	(1.59 -	10.07)	0.003
Exceed 60 mins	2.42	(0.35 -	16.86)	0.372
Exceed 120 mins	1.64	(0.10 -	26.52)	0.727
Others				
Exceed 10 mins	4.32	(0.90 -	20.73)	0.067
Exceed 30 mins	3.45	(0.59 -	20.25)	0.171
Exceed 60 mins				
Exceed 120 mins				

Relative to Hospitalist (Hospitalist OR: 1.00).
